# Quantitative results of SonoSpeech Cleft Pilot: a mixed-methods pilot randomised control trial of ultrasound visual biofeedback versus standard intervention for children with cleft palate ± cleft lip

**DOI:** 10.1186/s40814-025-01640-6

**Published:** 2025-05-06

**Authors:** Maria Cairney, Lisa Crampin, Linsay Campbell, Joanne Cleland

**Affiliations:** 1 University of Strathclyde, 50 George Street, Glasgow, G1 1QE UK; 2 NHS Greater Glasgow and Clyde, Royal Hospital for Children, 1345 Govan Road, Govan, Glasgow, G51 4TF UK

**Keywords:** Cleft palate ± cleft lip, Articulation intervention, Ultrasound visual biofeedback, Speech therapy

## Abstract

**Background:**

Despite its growing popularity, there is limited evidence of the effectiveness of ultrasound visual biofeedback speech therapy for children with cleft palate ± cleft lip (CP ± L). This study reports on the findings of a pilot feasibility study of ultrasound visual biofeedback versus standard care. Results will be used to determine if a full-scale randomised controlled trial (RCT) is feasible.

**Methods:**

We used a mixed-methods pilot RCT. Participants were children aged 5–16 with repaired CP ± L and at least one compensatory articulation. Participants were randomised, stratified for age, to receive six sessions of either articulation therapy (standard care) or ultrasound visual biofeedback (U-VBF) therapy. Outcome indicators for progression to full trial were measured as percentage targets achieved including the following: participants recruited and retained; outcome measure completion; and therapy protocol adherence. Due to the nature of treatment, the treating Speech and Language Therapists (SLTs) and families were not blinded; however, the assessing SLTs were blinded to treatment allocation until the end of the trial.

**Results:**

Eight participants were randomised to articulation therapy and eleven to ultrasound. All participants’ data was included for analysis. All but one of the pre-determined criteria for moving to full trial were fully met and the remaining indicator was partially met. At least 75% of the following were achieved: outcome measure completion; therapy protocol adherence; participant retention in each arm of the study. The target number of participants, 20 per treatment arm, was not reached.

**Conclusion:**

Most feasibility measures were successful. This study suggests that a full RCT comparing articulation therapy to U-VBF therapy would be possible if the current recruitment strategy is addressed.

**Trial registration:**

ISRCTN, ISRCTN17441953. Registered 22 March 2021.

## Key messages regarding feasibility

- The main uncertainties prior to this study were how easy it would be to recruit children and families to a trial and whether these families would be retained for the duration of the trial.
- Recruitment to the trial was challenging, but families who did join the trial completed it. All but one of the predetermined criteria for moving to full trial were met.
- A full-scale study will need to involve multiple cleft centres and offer a flexible number of therapy sessions until the target speech sounds are acquired.

## Background

Cleft palate ± cleft lip (CP ± L) is one of the most common congenital conditions in the UK [1, 2]. It is associated with speech difficulties even after successful surgeries to close the cleft [3]. These speech difficulties can be classified into two types: passive, resulting from anatomical differences (e.g., velopharyngeal insufficiency), or active, resulting from the speaker’s attempt to achieve the target sound with an alternative articulation, compensating for past or present anatomical differences [4]. Speech differences, associated with CP ± L, are often a subject to social stigma, which can have a negative psycho-social impact on the individual [5].

Active speech errors are differentially diagnosed from passive errors by Speech and Language Therapists (SLTs), who also provide speech therapy to address them. Currently, the prevalent SLT intervention approach for active oral speech errors is articulatory speech therapy, also known as articulation intervention or motor phonetic intervention [6]. Phonological approaches can also be used, and there is some evidence these are gaining in popularity [7]. Articulatory speech intervention is based on the principles of motor learning and involves drill-based practice of the target speech sounds in different speech contexts, gradually increasing in complexity. In more recent work, this is combined with “Knowledge of Performance” and “Knowledge of Results” feedback [8]. In contrast, phonological intervention focuses on the sound system that the child is acquiring and the child’s cognitive-linguistic understanding of differences between speech sounds, rather than on the motor execution of individual sounds [9].

One challenge of the articulatory approach is that it requires verbal explanations for how the tongue and other articulators should move in the oral cavity in order to achieve a specific speech sound. This can be problematic for some patients, particularly for younger children or those with language difficulties, which often co-occur in children with CP ± L [10, 11]. In addition, most of the tongue is hidden from view in the oral cavity, making it challenging for the SLT to use demonstration and difficult to avoid giving complex verbal explanations. This need for verbal explanations is also present in phonological interventions.

An alternative to these traditional speech interventions are visual biofeedback interventions. These interventions use articulatory instrumentation to show the child a real-time moving image representing the movement of their articulators. Most of the visual biofeedback evidence base in CP ± L focuses on electropalatography (EPG), which involves a custom-made artificial palate, fitted with electrodes [12]. EPG shows when and where the tongue makes contact with the hard palate in real time on a user-friendly computer display [13]. SLTs then encourage the child to use this visual biofeedback to change their articulations. Thanks to these advantages, EPG was part of the toolkit of many UK-based cleft specialists over the last two decades [14].

Despite these advantages, and a number of small studies showing evidence of successful EPG-based intervention, this approach has some serious drawbacks. First, the requirement for a custom-made palate leads to a high per-patient cost, long waiting times, and a limited time window to utilise the custom-made palate, as children grow and secondary dentition arrives or further surgery is required. Second, the EPG shows only the inferred position of the tongue, but it does not provide direct information about the actual shape and movement of the tongue. The tongue is capable of high levels of articulation [15, 16], and even if there is information about which part of the palate is touched, there is no information about which part of the tongue produced this contact. Third, related to the previous point, EPG cannot show uvular or pharyngeal articulations, which can be common speech errors in patients with CP ± L. These drawbacks, and in particular the high per-patient cost, are the reasons why most UK cleft SLT centres are transitioning away from using EPG in their therapy.

An alternative visual biofeedback tool, which avoids these disadvantages, is Ultrasound Visual Biofeedback (U-VBF). This tool uses standard medical ultrasound. The probe is placed in contact with the skin under the patient’s chin, showing a real-time moving image of the whole surface of the tongue in midsagittal view. The same system can be used on different patients, so there is no additional per-patient cost or waiting time, after the initial purchase of a system. There is a growing evidence base for the effectiveness of U-VBF in speech sound disorders in general [17], but a very limited number of studies have investigated its application with children with CP ± L-related speech difficulties. The small number of single case or case series studies which do use it show promising results [18]. A recent study used a case series design to show that five children with CP ± L had significant gains in percentage of targeted consonants correct when treated with U-VBF [19]. However, to date, no RCTs have compared U-VBF with either articulation or phonological therapy in this patient group. Despite the limited evidence base, UK cleft teams have been investing in the technology and some, such as NHS Glasgow and Clyde, have years of clinical expertise in the application of this intervention tool.

To address this urgent need for systematic investigation of the effectiveness of U-VBF for CP ± L-related speech difficulties, a randomised controlled trial (RCT) is required. However, an effective RCT requires a sample size analysis, which is normally carried out on the basis of data collected in a pilot studies or other intervention studies [20]. A feasibility RCT is also required to determine if the instrumental treatment, which involves a stabilising headset and an ultrasound probe with gel, will be acceptable to children of different ages. In addition, we need to determine if the process of randomisation will be acceptable to families, and whether they will be able to commit to all phases of a trial. Finally, before running a full-scale trial, it should be determined if the treating SLTs are able to adhere to the protocol and complete and administer all trial measures.

This paper reports on the results of a feasibility pilot RCT, which addresses these requirements by comparing U-VBF to articulatory therapy. We report the views of the children and their families who took part in the trial in a separate publication [21]. The protocol for this study was previously published in Cleland et al. [22].

### Objectives

We set success criteria, indicative that a full-scale RCT is warranted, as 75% and above, consistent with [20] for the following objectives:

1. To determine recruitment and attrition rates.

- 75% of children and their families identified agree to participate.
- 75% of children allocated in each group are retained for the duration of the study.

2. To measure pre-post and follow-up outcome measure completion.

- 75% of outcome measures are completed.

3. To measure within-session outcome measure completion.

- Data is reported from 75% of intervention sessions.

4. To determine acceptability of randomisation to children and their families.

- 75% of children and their families rate randomisation as acceptable in a questionnaire.

5. To determine the acceptability of ultrasound visual biofeedback as an assessment tool (both groups) and intervention tool (U-VBF group).

- 75% of children and their families rate ultrasound as an acceptable technique in a questionnaire.
- Focus group analysis contains more positive than negative themes regarding acceptability.

6. To measure adherence to the treatment protocol.

- 75% of sessions reach the minimum dosage of 100 trials in both treatment arms [22].

Aims 4 and 5 relating to acceptability were addressed in a separate publication [21].

## Methods

### Trial design

#### Description

This study used a mixed-methods two-arm parallel group pilot randomised controlled trial design. The study included a control condition, which was articulatory speech therapy acting as treatment as usual, and a U-VBF therapy arm. Participants in the control condition were offered the option of U-VBF therapy after the end of the trial if clinically appropriate. Children were randomised in a 1:1 ratio, stratified by age, within the following age groups (years;months): 4;6–7;11; 8;0–11;11; 12;0–16;0.

### Important changes to methods

Some minor changes of the protocol took place. First, the qualitative study (reported elsewhere in [21]) included a mix of interviews and focus groups, rather than focus groups alone, due to scheduling difficulties with the participants. Second, the planning stage of the study coincided with national travel restrictions due to the COVID-19 pandemic when travel for research assessments was not categorised as essential travel. Therefore, in setting up the study there was a contingency plan to carry out the baseline and follow-up assessments online via video conferencing software Microsoft (MS) Teams or Zoom. Due to challenges with recruitment, which were often related to difficulties with travel (see [21]), families were offered to participate in assessments remotely, which two families chose. All intervention took place in person.

### Eligibility criteria

Children on the caseload of the Scottish Cleft Lip and Palate service at NHS Greater Glasgow and Clyde were eligible for the study. Children and young people with any oral cleft-type, aged between 4;6 and 16;0 years, attending SLT clinics, were considered for participation and invited to the study. Children had to have at least one cleft-speech error that could benefit from either U-VBF or the articulatory intervention. The exclusion criteria were as follows: uncorrected bilateral hearing loss over 30 dB, based on past reports; any planned surgery in the following 3 months; or a severe language deficit, identified based on previous reports, or a British Picture Vocabulary Scale 3 (BPVS) standard score of < 70 during the baseline assessment [23].

Informed consent was obtained from all the families and children. We ensured that families had time to read and consider the participant information and have all their questions answered before the baseline eligibility screening. This eligibility screening was carried out by the research SLT (MC) who was not involved in the therapy. Screening for language abilities included the BPVS [23]. Screening for eligible speech errors that would benefit from either of the two study interventions was carried out using three assessments:

1. The phonology and articulation subtests of the Diagnostic Evaluation of Articulation and Phonology (DEAP) [24].
2. An ultrasound tongue imaging protocol, designed for a previous project [25]. The ultrasound tongue imaging protocol assessment included sentences from the GoS.SPaSS.’98/CAPS-A [26] and aimed to identify covert speech errors from consonants in all places of articulation. When assessments were carried out online, the same protocol was used but the audio-visual assessment was replaced by an audio-perceptual one.
3. Elicitation of a target-specific word list to assess the Percentage target Consonants Correct (PCC) before treatment. Children had to score <30 PCC at baseline to be eligible for the study.

### Settings and locations for data collection

This study was a single-centre study with all intervention sessions at the Royal Hospital for Children in Glasgow. The baseline and two follow-up assessments took place either in person at The University of Strathclyde in central Glasgow or online via MS Teams or Zoom.

### Interventions

Both interventions were administered by two experienced cleft specialist SLTs (LCr and LCa), working at the Royal Hospital for Children, Glasgow. The SLTs had completed training in using U-VBF therapy and were using it in their regular practice prior to the study. One or both SLTs were present during the therapy sessions, depending on availability. In sessions where both therapists were present, one delivered therapy and the other tracked the number of trials produced by the child. A minimum of 100 trials per session of the target speech sound were required in both intervention arms, in line with [27]. The therapists tallied the productions by hand as they occurred for fidelity checking.

Each participant received 6 sessions total: one per week, for 6 weeks. Each session lasted on average for 30 min with a range of between 20 and 40 min. Sessions were delivered one-to-one and in person in a quiet clinic room in the hospital. It was acknowledged before beginning intervention that this dosage was unlikely to lead to full generalisation. The number of sessions was chosen as anecdotally it is the number frequently offered within one SLT care of episode by the National Health Service (NHS) in this region, although there is no official standard number of sessions. We expected to see an initial response to intervention.

Treatment target wordlists were used during intervention, supplemented by personalised practice words, and participants were often encouraged to suggest words containing the target sound that were relevant to them, for example, the names of friends. The SLTs avoided using the words from the untreated wordlist during intervention.

### Articulation intervention

The articulation intervention focused on one sound at a time and was based on modelling, demonstration, verbal description and feedback from the SLT, particularly in the pre-practice stage [6]. Pre-practice refers to acquisition or learning the articulation of the speech sound in error. Practice refers to practising this new sound in different phonetic contexts. The sounds were introduced in limited phonetic contexts, in isolation or non-word monosyllables. This was to encourage participants to focus on the sound and tongue placement, rather than the phonemic category of the sound. Gradually, real words of increasing complexity and then phrases were introduced with the eventual aim of reaching conversation practice. The SLTs punctuated practice trials with one or two turns at an age-appropriate table-top game, chosen by the child.

### Ultrasound visual biofeedback (U-VBF)

The U-VBF intervention was based on the principles of motor learning. The participants saw a real-time moving midsagittal ultrasound image of their tongue displayed on a computer screen [28]. With the guidance of the SLT, the participants used this information to learn to associate the movement in their tongue with the changes of the image on screen. Similar to the articulatory intervention, the target sound was practised in increasingly complex contexts, initially dissociated from its phonemic category. Details of the intervention can be found in an open-access manual [29]. In this study, the software SonoSpeech [30] was used to display the ultrasound image to the participants. This software has been designed to support SLTs in speech assessment and therapy, including functionalities such as visual landmark icons, indicating correct and incorrect tongue placement, added to the ultrasound display. At the start of each session, the participants were encouraged to choose their preferred positive and negative landmark icons (e.g., a smiley face and a sad face, thumbs up and thumbs down).

### Outcomes

#### Rationale for outcome selection

The feasibility nature of this study meant that the measures are descriptive, focusing on the acceptability, implementation, and practicalities such as recruitment, retention, and measure completion rates [31]. Table 1 presents a list of the feasibility criteria alongside its analysis metric, method of aggregation, and time point of measurement.

**Table 1:** A list of the feasibility measures, its metrics and aggregation, and the time point of measurement

Pilot measures	Metric and aggregation as primary outcome measure	Time points
Recruitment rate	% participants of the target for each group are randomised	t0 (allocation)
Retention rate	% participants of those randomised completing at least one post-treatment measure	t8 (1-month post-treatment)
Completion of clinician-reported fidelity measure	% therapy sessions in each intervention where the dose tallies were was recorded	t1 to t6 (therapy)
Therapy protocol adherence	% therapy sessions in each intervention where the minimal dose was achieved	t1 to t6 (therapy)
Perceptual rating of treatment wordlists	% rated sessions	t(−1) and t1 to t8 (baseline, therapy, and post-treatment assessments)
Patient-reported outcome measure Cleft-Q (functioning and distress subscales)	% completed questionnaires for each intervention arm	t(−1), t7, t8 (baseline and both post-treatment assessments)
Patient-reported outcome measure ICS	% completed questionnaires for each intervention arm	t(−1), t7, t8 (baseline and both post-treatment assessments)
Patient-reported experience measure	% completed questionnaires for each intervention arm	t8 (1-month post-treatment)
Intervention acceptability questionnaire	% completed questionnaires for each intervention arm	t8 (1-month post-treatment)

There were no continuous variables analysed as categorical. In this pilot, within-participant change is only reported descriptively, using plots and/or the aggregates listed in Tables 1 and 2.

**Table 2:** A list of the candidate primary and secondary outcome measures, their metrics, and aggregation at the time point of measurement

**Candidate primary outcome measure**	**Metric and aggregation as primary outcome measure**	**Time points**
Perceptual ratings by blinded assessors of target consonants in treated and untreated wordlists	% target Consonants Correct (PCC)	t(−1), t7, t8 (baseline and both post-treatment assessments)
Perceptual ratings by blinded assessors of target consonants of within-treatment wordlists	% target Consonants Correct (PCC)	t1 to t6 (therapy)
**Candidate secondary patient-reported outcome measures**	**Metric and aggregation as primary outcome measure**	**Time points**
Patient-reported outcome measure Cleft-Q (functioning and distress subscales)	Standard score	t(−1), t7, t8 (baseline and both post-treatment assessments)
Patient-reported outcome measure ICS	Standard score	t(−1), t7, t8 (baseline and both post-treatment assessments)
Patient-reported experience measure	% positive, negative and neutral responses	t8 (1-month post-treatment)
Intervention acceptability questionnaire	% positive and negative responses	t8 (1-month post-treatment)

As this is a pilot feasibility study, minimal important change in individuals was not determined in advance. The results from this study and other recent cases series of U-VBF will be used to inform future full-scale iterations of this study [19]. Six sessions were chosen as a feasible number within the confines of the health service. However, this number is likely to be an insufficient dosage to lead to clinically significant change [17, 32]. Instead, we expected participants to show evidence of initial response to treatment and we anticipate future trials should consider dosage carefully.

### Study instruments

The Intelligibility in Context Scale (ICS) [33] was used as a carer-reported outcome measure. It has high internal reliability and construct validity, particularly with younger children and children with CP ± L [33–35]. The Cleft-Q speech function and distress scales were used as a quality-of-life measure [36]. The scales were completed by children aged 8 and over and by carers of children younger than 8. This measure has been tested internationally using large and diverse samples, and it was determined to have good content and construct validity, as well as good reliability [36]. The Experience of Service Questionnaire (ESQ) [37] measured the parent/carer satisfaction with the interventions and was administered at the end of participation. It has good construct validity and precision [37]. The questionnaire scores were calculated by the research SLT, according to manual instructions. The perceptual ratings of PCC were carried out by eight CAPS-A [38] trained cleft specialist SLTs across the UK who were blinded to the participants’ intervention allocation, participant number, and time point of recording.

Data quality was promoted during questionnaire data collection by providing sufficient time for completion and prompting carers and children to ask questions if anything was unclear. Data quality was promoted when recoding audio and ultrasound video by following a checklist and by taking note of interfering factors (e.g., child moving excessively). Data quality was promoted when eliciting perceptual ratings by the specialist SLTs by providing the same instructions to all.

### Changes to trial outcomes after trial commenced with reasons

Due to an error of the research SLT, the target and generalisation probe used in the baseline, therapy, and follow-up assessments did not include prompts to produce the target consonants in isolation and in sentence contexts. It did however include syllables, non-words, and real words of increasing complexity, which we deem sufficient for measuring progress.

### Sample size

The target number of participants was 20 per intervention arm. This was chosen heuristically as a feasible number for a pilot study. Given the 75% retention target to assess feasibility, the margin of error would be approximately ± 18% (95% Confidence Interval, 53 to 89%) (Wilson score interval). No interim analyses were carried out, as per protocol, and recruitment stopped before the target number was achieved due to time constraints.

### Randomisation and blinding

Eligible consenting participants were randomised by the Glasgow Clinical Trials Unit in a 1:1 ratio, stratified for age using an electronic platform. The participants were stratified into the following age groups: 4;6–7;11; 8;0–11;11; 12;0–16;0. The participants were enrolled by the research SLT who was blind to their allocation until the trial finished. The treating SLTs were notified by an email generated by the online system of the participants’ allocation. The specialist SLTs who carried out the perceptual analysis of audio recordings of the target list productions were also blinded to the participants’ allocation.

### Statistical methods

Descriptive analyses were used for the analysis of the primary outcome measure’s mean percentages and standard deviations. When calculating Percentage target Consonants Correct (PCC), all transcribers marked word-medial glottal realisations of /t/ as incorrect for children working on /t/-targets. This is a typically occurring variant in Scottish accents, which all children had, so PCC was re-calculated by the first author to mark it as correct in the relevant wordlists.

Missing data is described in the Results section as it is linked to our primary outcomes. There is no multiplicity of analyses. Some data were recorded incorrectly and had to be excluded. For some participants, the wrong therapy target was assessed at baseline and/or follow-up due to experimenter error, so this audio assessment data was excluded: participant 7 (ultrasound group; baseline and 2 follow-ups), participant 11 (ultrasound group; baseline), participant 14 (articulation group; baseline and follow-up 1), participant 15 (articulation group; baseline and 2 follow-ups) (Table 3). For participant 18, the wrong therapy target word list was assessed at Therapy session 6, which was excluded as well. Reflections on these errors are discussed in the Limitations section.

Data wrangling was carried out using the “tidyverse” (version 2.0.0) [39] package in R (version 4.3.1) and R Studio (version 2023.12.0 + 369) [40]. Data visualisation was carried out using “ggplot2” (version 3.4.3) [41] and “ggpubr” (version 0.6.0) [42] packages, and ICC calculations were made using the “psych” (version 2.4.1) [43] package. The data and code are available via https://osf.io/gkd3h/?view_only=1543a2b027fd4d7aaa0b6a70d176863a.

## Results

### Participant flow

#### Recruitment

##### Dates defining the periods of recruitment and follow-up 

The first participant was recruited on 26 November 2021 and the final participant was recruited on 4 May 2023. Follow-ups were performed for each participant within one week and one month after the final therapy session. The final follow-up took place on 26 July 2023.

One child with BPVS standard score of < 70 was admitted to the study, due to the clinical decision of the treating SLTs who had offered successful treatment to this patient in the past.

#### Trial end

The trial was extended by 6 months with agreement from the funder due to staffing changes. Due to challenges with participant recruitment, potential participants were approached until September 2023, 6 months before the end of the trial.

### Baseline data

**Table 3:** Participant baseline demographic and clinical characteristics [21]

*Participant ID*	*Treatment*	*Target consonant*	*Age*	*Sex*	*Additional diagnoses*	*Language spoken at home*	*Other languages*	*Years in Scotland*	*Accents spoken at home*	*Type of cleft*
*SS_01_F_07*	Ultrasound	/t/	7;4	F	n.a.	English	n.a.	7	Northeast Scottish and Glaswegian	UCLP
*SS_02_M_12*	Ultrasound	/s/	12;11	M	n.a.	Portuguese, English	French (not fluent)	12	Portuguese	BCLP
*SS_03_M_07*	Articulation	/t/	7;3	M	n.a.	English	n.a.	7	Scottish	CP
*SS_04_M_05*	Ultrasound	/t/	5;5	M	n.a.	English	n.a.	5	Scottish	UCLP
*SS_05_M_05*	Articulation	/s/	5;7	M	n.a.	English	n.a.	5	Scottish	UCLP
*SS_06_F_09*	Ultrasound	/t/	9;10	F	n.a.	English, Filipino	(Not reported)	(Not reported)	(Not reported)	UCLP
*SS_07_M_08*	Ultrasound	/t/d	8;9	M	n.a.	English	n.a.	8	Scottish	UCLP
*SS_08_M_07*	Ultrasound	/k/	7;6	M	Asthma	English	n.a.	3	Scottish	Submucous cleft
*SS_09_M_15*	Ultrasound	/t/	15;6	M	n.a.	English	n.a.	15	Scottish	BCLP
*SS_10_F_05*	Articulation	/s/	5;2	F	n.a.	English	n.a.	5	Scottish	CP
*SS_11_M_07*	Ultrasound	/t/	7;6	M	n.a.	English	n.a.	6	Scottish	BCLP
*SS_12_F_05*	Ultrasound	/s/	5;0	F	n.a.	English	n.a.	5	Scottish	BCLP
*SS_13_F_05*	Articulation	/t/	5;8	F	Stickler Syndrome, hearing loss	English	n.a.	5	Mum Scottish, dad Geordie	CP
*SS_14_F_04*	Articulation	/s/	4;6	F	Stickler Syndrome, hearing loss	English	n.a.	4	Mum Scottish, dad Geordie	CP
*SS_15_M_07*	Articulation	/t/	7;11	M	Hearing impairment	English	n.a.	7	Scottish	UCLP
*SS_16_M_06*	Ultrasound	/t/	6;5	M	n.a.	English	n.a.	6	Scottish	CP
*SS_17_F_10*	Articulation	/t/	10;4	F	n.a.	English	n.a.	10	Scottish	UCLP
*SS_18_M_11*	Articulation	/t/	11;9	M	22q11 deletion syndrome	English	n.a.	11	Mum/brother Scottish, dad Bristolian	CP
*SS_19_M_11*	Ultrasound	/s/	11;8	M	n.a.	English	n.a.	11	Mum Scottish, dad English	BCLP

### Outcomes and estimation and numbers analysed

#### Feasibility measures

Table 4 summarises the feasibility measures results. The pre-determined criteria of at least 75% completion of each measure were met for all bar one important target—the recruitment rate.

**Table 4:** Primary feasibility outcomes presented as proportions and percentages

Analysis	Articulation therapy		U-VBF therapy	
	%	Proportions	%	Proportions
Recruitment rate	40	8/20	55	11/20
Retention rate	100	8/8	90.9	10/11
Baseline PCC	75	6/8	80.8	9/11
Baseline CleftQ	87.5	7/8	100	11/11
Baseline ICS	100	8/8	100	11/11
Follow-up 1 PCC	75	6/8	90.9	10/11
Follow-up 1 CleftQ	100	8/8	90.9	10/11
Follow-up 1 ICS	100	8/8	90.9	10/11
Follow-up 2 PCC	87.5	7/8	80.8	9/11
Follow-up 2 Cleft Q	87.5	7/8	90.9	10/11
Follow-up 2 ICS	87.5	7/8	90.9	10/11
Follow-up 2 ESQ	100	8/8	100	11/11
Follow-up 2 Intervention acceptability questionnaire	87.5	7/8	90.9	10/11
Therapy session 1 PCC completed	100	8/8	81.8	9/11
Therapy session 2 PCC completed	75	6/8	90.9	10/11
Therapy session 3 PCC completed	100	8/8	100	11/11
Therapy session 4 PCC completed	100	8/8	100	11/11
Therapy session 5 PCC completed	87.5	7/8	80	8/10
Therapy session 6 PCC completed	75	6/8	80	8/10
Therapy sessions, reaching dose 100	100	48/48	98.4	63/64
Within-therapy session completed dose recording	87.5	42/48	96.9	62/64

Our definition for retention was for participants to complete all therapy session and return for at least one follow-up. One participant in the articulation did not return to one follow-up due to bereavement but returned to the first follow-up, so they counted as retained. One participant in the U-VBF group missed the first follow-up due to an overseas trip, but they self-recorded a production of the target word-list, which was included in analyses. One participant in the U-VBF group was discontinued by the treating SLTs after 4 sessions because of wellbeing concerns unrelated to the study; however, they returned for one follow-up session. They are counted as not retained because they did not complete all therapy sessions, but all their available data is included in analyses.

Table 5 summarises the percentage outcomes for the candidate primary and secondary outcome measures, which will be used in a full-scale clinical trial.

**Table 5:** Candidate primary and secondary outcome measures: percentage/proportion (standard deviation) and numbers of participants analysed

Candidate primary outcome: Percentage targeted consonants correct, measured as the percentage of treated speech sounds produced correctly in words, using therapists’ auditory assessment		
Time point	Articulatory therapy	U-VBF therapy
Baseline	13 (33), *n* = 6	14 (35), *n* = 9
Therapy session 1	16 (37), *n* = 8	19 (39), *n* = 9
Therapy session 2	21 (41), *n* = 6	25 (43), *n* = 10
Therapy session 3	27 (44), *n* = 8	14 (35), *n* = 11
Therapy session 4	26 (44), *n* = 8	14 (35), *n* = 11
Therapy session 5	26 (44), *n* = 7	29 (46), *n* = 8
Therapy session 6	12 (32), *n* = 6	21 (41), *n* = 8
1 week after intervention	43 (50), *n* = 6	22 (41), *n* = 10
1 month after intervention	39 (49), *n* = 7	37 (48), *n* = 9
Carer-reported intelligibility for children, measured using the Intelligibility in Context Scale		
Baseline	0.72 (0.08), *n* = 8	0.75 (0.12), *n* = 11
1 week after intervention	0.76 (0.09), *n* = 8	0.76 (0.12), *n* = 10
1 month after intervention	0.71 (0.05), *n* = 7	0.78 (0.08), *n* = 10
Patient- and carer-reported quality of life, measured using the CLEFT-Q speech function scale		
Baseline	54.57 (7.35), *n* = 7	50.45 (15.40), *n* = 11
1 week after intervention	60.25 (9.44), *n* = 8	53.90 (14.87), *n* = 10
1 month after intervention	53.86 (9.08), *n* = 7	61.60 (16.79), *n* = 10
Patient- (aged ≥ 8) and carer-reported (aged < 8) quality of life, measured using the CLEFT-Q speech distress scale		
Baseline	72.43 (15.28), *n* = 7	66.00 (15.26), *n* = 11
1 week after intervention	76.57 (16.88), *n* = 7	70.00 (16.19), *n* = 9
1 month after intervention	77.86 (14.37), *n* = 7	70.40 (19.18), *n* = 10
Patient and carer satisfaction with both interventions, measured using the Experience of Service Questionnaire 1 month after intervention		
1 month after intervention	Results reported in [21]	

**Fig. 1:**
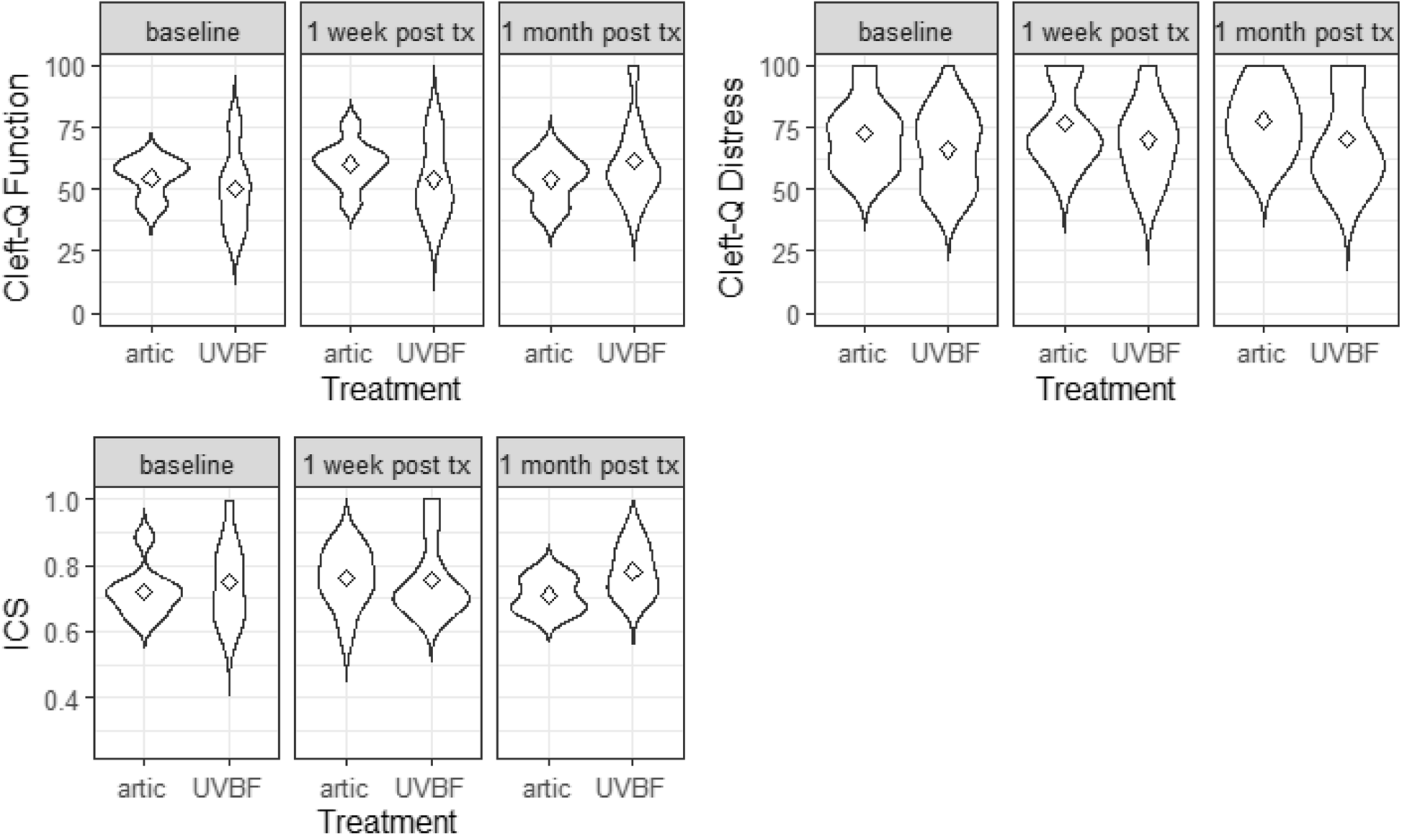
Violin plot and means of Cleft-Q and ICS results before, 1 week after and 1 month after therapy for each treatment group

Figure 1 illustrates the changes in the ICS and Cleft-Q subscales for children in each treatment arm across the three time points: baseline, 1 week after therapy, and 1 month after therapy.

### Ancillary analyses

#### Prespecified: harms and unintended consequences

No harms or unintended effects were reported. Participant 17 in the U-VBF condition temporarily overgeneralised an intermediate interdental target to other syllable-onset consonants. The treating SLTs used an interdental target for both groups to stimulate anterior placement of backed /t/ and gradually shaped it into the target alveolar /t/.

#### Prespecified: dose fidelity

Dosage of target consonant trials during therapy was recorded, as reported in Table 4. To measure fidelity, the therapy sessions were recorded, and a research SLT listened to a pseudo-randomly selected 20% of the data and tallied the number of target trials achieved. We ensured that at least one recording was randomly selected from each participant. Based on [44], an interclass correlation coefficient was calculated, using a two-way agreement single rater model. The results indicated a high level of agreement (ICC = 0.83, *F* (21,21) = 10.9, *p* < 0.001). It should be noted that in most cases, the SLTs providing therapy underestimated the number of trials the child produced (see Table 6 in the Appendix).

#### Prespecified: PCC agreement

The audio files of the target consonant wordlists were assigned to eight transcribers in order to calculate PCC, with 20% overlap in the files between pairs of transcribers. We calculated the ICC based on the raters’ per-session PCC results. We present the results of the average fixed raters’ model, because, where more than one rating was available, we wanted to use the average rating within our results and not the rating of a single rater. We also had a fixed set of raters, as opposed to a random population. The results indicate excellent agreement (ICC = 0.95, *F* (146, 1022) = 26, *p* < 0.001) [44]. However, because there was only 20% overlap and most of the PCC results were available from only one transcriber, we decided it is pertinent to also report that the single fixed raters’ model, which indicated good agreement between the raters (ICC = 0.76, *F* (146, 1022) = 26, *p* < 0.001) [44].

#### Exploratory: responders and non-responders

An exploratory plot is presented in 2 showing the PCC per session per participant. Out of 19 participants, ten can be described as responders, as their follow-up scores are higher than their baselines scores: 3, 5, 13, 17, and 18 in the articulation group and 4, 7, 8, 11, and 19 in the ultrasound group. Participants 1, 2, 12, 16 (U-VBF), and 10 (articulation) showed almost no change in their PCC across the sessions and can be classified as non-responders within the limited six-session episode of care. The remaining participants achieved some change in their articulation as a result of the therapy, even if it did not result in accurate target consonant realisations in the follow-up measurements. The discussion will revisit these findings, in light of the limitations of PCC as a measure of intermediate progress and having only 6 therapy sessions.

## Discussion

### Summary of findings

**Fig. 2:**
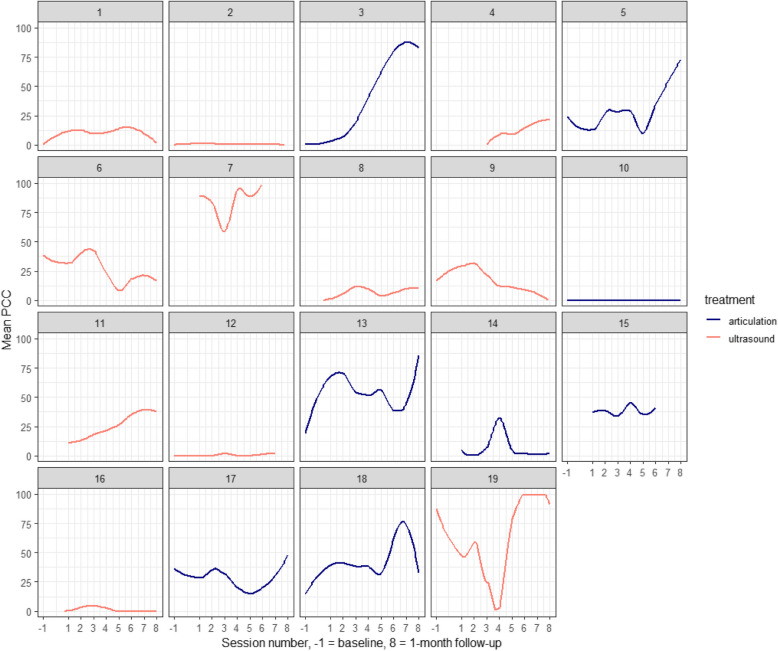
Exploratory plot showing the mean PCC per participant per session, including the baseline as -1, therapy sessions 1–6 and the two follow-up sessions as 7 and 8.

This study aimed to test the feasibility of a RCT comparing U-VBF to articulatory intervention in children with CP ± L. To our knowledge, this is the first feasibility RCT comparing U-VBF to articulatory therapy for children with CP ± L. The results suggest that the study is feasible, with some caveats. All but one of the pre-determined criteria for moving to full trial were met. While it was challenging to recruit the necessary number of participants, all participants who were enrolled in the study provided positive feedback of the experience [21] and almost all were retained until the end. Those who did not return for follow-up had serious personal circumstances which prevented them from doing so. It was feasible to collect at least 75% of each of the outcome measures at each of the relevant time points for each of the intervention arms (see Table 4). In addition, Table 5, illustrated in Figs. 1 and 2, shows positive trends for the speech, intelligibility, and quality of life outcome measures.

### Recruitment

One of the important findings of this trial was that it was challenging to recruit the target number of participants. The reasons for under-recruitment are addressed in depth in [21]. The COVID-19 pandemic had an impact on recruitment as some restrictive measures were still in place when the trial started, making travel more challenging. The cost of travel (although this was reimbursed for up to £10 per trip) and child-care, linked to the cost-of-living crisis, may have prevented some families from participating. Those that did engage in the trial reported that the time burden of travel was also significant. Competing health priorities for the children that were unaddressed during lockdown were also barriers to participation. Moreover, a move to online therapies during the pandemic may have made families reluctant to travel for a treatment which was offered in-person only. The treating SLTs had the main responsibility for recruitment. Due to their close familiarity with their patients’ history, they applied stricter selection criteria before approaching potential participants, particularly when they were familiar with previous history of non-engagement or complex family circumstances. While stricter criteria were applied in most cases, it was decided that an older child with language abilities below the threshold standard score of 70 on the British Picture Vocabulary Scale [23] could benefit from treatment. This decision was made in light of the fact that younger children with language abilities that were equivalent, albeit typical for their age, were considered eligible. Due to the high co-occurrence of language difficulties and CP ± L [10, 11], future studies should consider applying raw score, as opposed to standard score, thresholds for eligibility. Despite not reaching the 75% recruitment target, according to [20], 40–80% success rate, which this study falls within, should be considered for moving to full trial. Future trials can mitigate recruitment issues by having multiple centres, more choice of intervention venues, and by approaching a wider group of participants at the trial outset.

### Retention and speech assessment completion

The retention rate of this study was high, similar to [45], which may be a result of the high scrutiny at the selection stage in our study. Only one participant was discontinued from the intervention due to wellbeing concerns, unrelated to the speech intervention. However, they returned for one follow-up assessment. Another participant did not return for the final assessment due to bereavement. All speech assessment data collection targets were met, despite some challenges that arose from having multiple candidate speech targets for intervention in some children and difficulties in communicating them to the treating clinicians. Future trials can address these challenges by formalising the procedure of recording the therapy target and ensuring regular communication with the SLTs. All transcriptions of the speech samples were undertaken by blinded assessors. The percentage target consonants correct (PCC) ratings suggest improvement in the speech accuracy of the participants, consistent with other reports on the effectiveness of U-VBF therapy in CP ± L patients [19].

### Patient and carer-reported outcome measures

Unlike other feasibility trials, such as [45], this study surpassed its targets for questionnaire completion. This may be attributed to the fact that most participants were assessed in person at the research centre, where there was dedicated space and time for participants and carers to complete the questionnaires. However, one limitation of this approach is that for some participants, the questionnaires were completed by different carers at different time points in the study, which could introduce some variability in the responses. Future studies can mitigate this issue by mailing hard copies of the questionnaires to a dedicated family member at home, in addition to giving a questionnaire to the carer who accompanies the child to the assessments. As reported in [21], the patient and carer experiences of the ultrasound intervention were overall positive, indicating high satisfaction and acceptability of the treatment. Moreover, visual observation of Fig. 1 also shows a trend for improvement in the scores of the outcome measures in children who received the ultrasound condition. This is particularly the case for the Intelligibility in Context Scale (ICS) and the Speech Function subscale of the Cleft-Q, suggesting that the intervention had a positive impact on their speech and quality of life. This is consistent with [32] where visual biofeedback speech therapy was provided to people with CP ± L.

### Clinician-reported fidelity measures

The SLTs achieved the minimum of 75% dose recording during sessions (i.e., recording at least 75% of the target consonant repetitions). Although in many cases the SLTs worked on their own, occasionally they worked in a pair, allowing one of them to focus on delivering therapy and the other to tally the target consonant repetitions. The agreement between their tallies and the research SLT's were high, although the treating SLTs often underestimated the number of consonant repetitions. One factor that may have contributed is that the children often spontaneously produced more practice trials, which were not prompted by the treating SLT and may have therefore been more challenging to record. The treating SLTs also surpassed the threshold of audio recording a minimum of 75% of therapy sessions, to allow for PCC measurement and fidelity checking. The two treating SLTs were experienced with research, which may have contributed to their high success rate. Future trial managers need to ensure that all SLTs across all participating centres are able to follow the same therapy preparation checklist by providing training.

### Limitations

A key limitation of this study is that the sample size was chosen heuristically, instead of using a formal calculation to determine a sample size that would allow estimating feasibility parameters to an acceptable level of precision. This, together, with the limited recruitment will have implications for the accuracy of future sample size calculations.

In addition, minimal clinically important changes were not pre-specified. It has recently been recommended that minimal clinically important differences are better determined from the literature, rather than feasibility studies [46]. At the time of planning this project, there was a lack of studies comparing UVBF therapy for children with CP ± L to other therapies. Therefore, we previously anticipated that the results of this study would inform future decisions about clinically significant change and sample size. We note that since then there has been one other study investigating UVBF with this population, which can be used to inform sample size calculations [19].

An additional limitation of this study was the cumulative intervention intensity. The target dose was a minimum of 100 teaching episodes per intervention session, with one 20–30-min intervention session per week for 6 weeks. This led to a minimum cumulative intervention intensity of 600 teaching episodes (not including any practice at home), which, along with the dose frequency, is considered low in the context of speech interventions. This is lower than, for example [8]. They reported clinically significant improvement with a cumulative intervention intensity of 1600 per phase of intervention, delivered in two phases for a total of 3200 for children with CP ± L. It needs to be noted, however, that the treating SLTs in the present study often achieved a higher number of teaching episodes per session, with an average of 223 (SD = 68), leading to an actual average cumulative intensity of 1338. Even so, the cumulative intensity in [8] is more than two times higher than in the present study. As discussed in the rationale for outcome selection, this number of sessions was chosen as a feasible target within the constraints of the publicly funded national health service, with the intention of producing initial articulatory changes. It is common for paediatric SLT services to offer children a maximum of six intervention sessions in a block, despite the evidence suggesting most interventions require a higher intensity. This is due to financial constraints within the publicly funded healthcare systems. However, participants remained on the SLTs’ caseloads and were offered further episodes of care after the end of their participation in the study, as needed. Progress achieved over six therapy sessions is considered rapid [32] and 6 weeks of once per-week intervention is often insufficient to achieve and generalise correct articulation [17, 32]. We suggest that a future RCT offers a flexible approach to cumulative intervention intensity, by holding the in-session dosage constant at least 100 trials per session but increasing the number of sessions flexibly until children achieve generalisation to untreated items. This is similar to the method used by [32] where participants had between 15 and 33 sessions of biofeedback intervention as required. Required dosage is likely to vary considerably between patients due to factors such as age, previous intervention history, and stimulability of intervention target.

Another limitation of the trial is the use of percentage target consonants correct (PCC) as a candidate outcome measure, as it may be a conservative estimate of progress, particularly across a limited number of sessions. The treating SLTs’ short-term targets for most participants included achieving forward tongue tip placement. This short-term target is observed in a number of children at the end of therapy (e.g., participant 9); however, a binary analysis of correctness would categorise these productions as incorrect, underestimating the effects of therapy. [47] report that almost all speech errors of people with CP ± L treated during their study showed a gradient, as opposed to a categorical, pattern of improvement. That paper also reports that an instrumental articulatory assessment was able to provide more complete and accurate information on the gradient realisations, compared to narrow phonetic transcription. A future articulatory analysis using the ultrasound tongue imaging data would be possible, and indeed previous work has shown gradient acquisition can be measured using this technique [48]. Where children showed early signs of gradient change during intervention, this should be considered a positive prognostic indicator that additional intervention is likely to be beneficial.

There was experimenter error, which led to differences between the consonant target recorded during baseline and/or follow-up assessments and the consonant target treated during therapy for some participants. This resulted from email communications being missed between the research and treating SLTs, as well as a lack of centralised database where treatment targets could be recorded. The impact of this error is mitigated by the audio recordings of the target and generalisation probes during therapy, which can demonstrate immediate effects of therapy. Another mitigation is that a general assessment wordlist, based on the CLEFTNET protocol, was recorded at all baseline and follow-up assessments, containing the target consonant [14, 29], which can be used for analysis in future investigations of this dataset. A future full-scale RCT should consider modifying the Trial Unit’s randomisation webpage to include the target consonant for therapy alongside the participants’ treatment allocation and age group.

Finally, only one cleft centre was included in this trial. This centre may not be representative of other centres across the UK as it is a cleft centre where U-VBF therapy was already offered prior to the trial. The treating SLTs were potentially advocates of this type of therapy and in some cases, they struggled to ethically justify approaching participants for whom they considered that U-VBF would outperform articulation therapy, based on their clinical expertise and the patients’ previous non-response to articulatory therapy. A recent qualitative study of SLTs’ perspectives on taking part in a clinical trial of U-VBF showed that clinicians in other cleft services were enthusiastic about recruiting to a trial, but shared concerns about randomising patients to interventions which they felt may not be in the patients’ best interests [49]. This can be resolved in a future trial by ensuring that all patients approached are suitable for both interventions and including centres who are new to the technique, or employing a cluster randomised control design where centres offer either ultrasound or treatment as usual only.

### Applicability of the trial findings

Due to the pilot nature of the study, the results cannot be considered generalisable. However, these findings will be used to plan a large-scale RCT comparing U-VBF to articulation intervention for children with CLP. This study has provided valuable insight into the challenges and opportunities of running an RCT in collaboration with NHS cleft centres.

## Conclusion

In conclusion, this study shows that a future full-scale RCT project comparing U-VBF to articulatory intervention in children with CP ± L is feasible with some caveats. Almost all of our criteria for a move to full trial were met: participants who were recruited to the study were retained, outcome measure completion was feasible, and both interventions were well-tolerated by the children and their families. Separate qualitative work [21] showed that participants enjoyed taking part in the study and that the ultrasound intervention in particular was enjoyable and enabled participants to gain new insights into the nature of their speech disorder. However, the burden of travel for participants was high, and this in turn may have led to some difficulties recruiting participants. This may be because despite the city centre location of our study, the cleft service covers a wide geographical region including remote and rural areas. Moreover, descriptive statistics suggest that in both intervention arms six sessions of intervention is inadequate dosage to lead to generalisation of targets. However, a move towards correct anterior productions was seen in most participants, suggesting that increased dosage would lead to better generalisation [32]. A future trial should consider offering more choice of intervention locations and a flexible dosage suited to each participant.

## Data Availability

Numeric anonymised data and statistical codes are available from the Open Science Framework (https://osf.io/gkd3h/?view_only=1543a2b027fd4d7aaa0b6a70d176863a). All bespoke data collection forms, such as the participant information sheets and the consent form templates, interview schedule and questionnaires, can be found in a publicly accessible folder on the University of Strathclyde open access Knowledge Base “PURE” (via DOI 10.15129/f65343c4-7781-44fe-9b00-516d4597efac).

## Appendix

**Table 6:** PCC per participant per session. *Ax* assessment, *Tx* therapy, *SD* standard deviation

Treatment	Participant	Session type	Session number	PCC	SD
Articulation	3	Ax	1	0	0
Articulation	3	Ax	2	89.58	30.87
Articulation	3	Ax	3	82.22	38.66
Articulation	3	Tx	1	5.88	23.88
Articulation	3	Tx	2	5.26	22.53
Articulation	3	Tx	3	22.06	41.77
Articulation	3	Tx	4	41.18	49.96
Articulation	5	Ax	1	25	43.76
Articulation	5	Ax	2	87.76	33.12
Articulation	5	Ax	3	62.89	48.56
Articulation	5	Tx	1	9.26	29.26
Articulation	5	Tx	2	7.41	26.44
Articulation	5	Tx	3	55.56	50.16
Articulation	5	Tx	4	0	0
Articulation	5	Tx	5	38.89	48.98
Articulation	5	Tx	6	0	0
Articulation	10	Ax	1	0	0
Articulation	10	Ax	2	0	0
Articulation	10	Ax	3	0	0
Articulation	10	Tx	1	0	0
Articulation	10	Tx	2	0	0
Articulation	10	Tx	3	0	0
Articulation	10	Tx	4	0	0
Articulation	10	Tx	5	0	0
Articulation	10	Tx	6	0	0
Articulation	13	Ax	1	19.57	40.11
Articulation	13	Ax	2	17.39	38.76
Articulation	13	Ax	3	95.65	20.85
Articulation	13	Tx	1	68.57	47.1
Articulation	13	Tx	2	70.59	46.25
Articulation	13	Tx	3	58.82	49.96
Articulation	13	Tx	4	37.68	48.81
Articulation	13	Tx	5	70.18	46.16
Articulation	13	Tx	6	41.18	49.96
Articulation	14	Ax	3	2.04	14.29
Articulation	14	Tx	1	4.9	21.7
Articulation	14	Tx	3	7.41	26.31
Articulation	14	Tx	4	32.41	47.02
Articulation	14	Tx	5	4.63	21.11
Articulation	14	Tx	6	1.85	13.61
Articulation	15	Tx	1	37.14	49.02
Articulation	15	Tx	2	38.24	49.33
Articulation	15	Tx	3	33.82	47.66
Articulation	15	Tx	4	44.93	50.11
Articulation	15	Tx	5	35.29	48.51
Articulation	15	Tx	6	41.18	49.96
Articulation	17	Ax	1	37.5	49.45
Articulation	17	Ax	2	27.66	45.22
Articulation	17	Ax	3	48.94	50.53
Articulation	17	Tx	1	20.59	41.04
Articulation	17	Tx	2	32.35	47.49
Articulation	17	Tx	3	38.24	49.33
Articulation	17	Tx	4	13.24	34.14
Articulation	17	Tx	5	17.65	38.41
Articulation	17	Tx	6	20.59	41.04
Articulation	18	Ax	1	14.29	35.86
Articulation	18	Ax	2	91.67	28.23
Articulation	18	Ax	3	26.09	44.9
Articulation	18	Tx	1	41.18	49.96
Articulation	18	Tx	3	35.29	48.51
Articulation	18	Tx	4	38.24	49.33
Articulation	18	Tx	5	26.47	44.45
Ultrasound	1	Ax	1	0	0
Ultrasound	1	Ax	2	8.7	28.81
Ultrasound	1	Ax	3	2.17	14.74
Ultrasound	1	Tx	1	14.71	35.95
Ultrasound	1	Tx	2	14.71	35.95
Ultrasound	1	Tx	3	2.94	17.15
Ultrasound	1	Tx	4	14.71	35.68
Ultrasound	1	Tx	6	14.71	35.95
Ultrasound	2	Ax	1	0	0
Ultrasound	2	Ax	2	0	0
Ultrasound	2	Ax	3	0	0
Ultrasound	2	Tx	1	1.85	13.61
Ultrasound	2	Tx	2	0	0
Ultrasound	2	Tx	3	0.93	9.62
Ultrasound	2	Tx	4	0	0
Ultrasound	2	Tx	6	0.93	9.62
Ultrasound	4	Ax	1	0	0
Ultrasound	4	Ax	2	20	40.41
Ultrasound	4	Ax	3	21.74	41.7
Ultrasound	4	Tx	3	0	0
Ultrasound	4	Tx	4	12.28	33.11
Ultrasound	4	Tx	5	8.93	28.77
Ultrasound	4	Tx	6	14.71	35.95
Ultrasound	6	Ax	1	39.13	49.9
Ultrasound	6	Ax	2	21.74	41.7
Ultrasound	6	Ax	3	16	37.03
Ultrasound	6	Tx	1	27.94	45.2
Ultrasound	6	Tx	2	29.41	46.25
Ultrasound	6	Tx	3	55.17	50.17
Ultrasound	6	Tx	4	19.12	39.62
Ultrasound	6	Tx	5	0	0
Ultrasound	6	Tx	6	26.47	44.45
Ultrasound	7	Tx	1	88.57	32.28
Ultrasound	7	Tx	2	82.86	38.24
Ultrasound	7	Tx	3	58.82	49.96
Ultrasound	7	Tx	4	94.29	23.55
Ultrasound	7	Tx	5	88.57	32.28
Ultrasound	7	Tx	6	100	0
Ultrasound	8	Ax	1	0	0
Ultrasound	8	Ax	2	16.67	37.55
Ultrasound	8	Ax	3	7.58	26.66
Ultrasound	8	Tx	1	0	0
Ultrasound	8	Tx	2	5.48	22.92
Ultrasound	8	Tx	3	10.81	31.26
Ultrasound	8	Tx	4	13.51	34.66
Ultrasound	8	Tx	5	0	0
Ultrasound	8	Tx	6	5.41	22.92
Ultrasound	9	Ax	1	17.39	38.76
Ultrasound	9	Ax	2	4.35	20.62
Ultrasound	9	Ax	3	0	0
Ultrasound	9	Tx	1	26.47	44.45
Ultrasound	9	Tx	2	29.41	46.25
Ultrasound	9	Tx	3	27.78	45.43
Ultrasound	9	Tx	4	4.41	20.69
Ultrasound	9	Tx	5	17.65	38.7
Ultrasound	11	Ax	2	33.33	48.15
Ultrasound	11	Ax	3	39.13	49.9
Ultrasound	11	Tx	1	14.71	35.95
Ultrasound	11	Tx	2	2.94	17.15
Ultrasound	11	Tx	3	23.53	43.06
Ultrasound	11	Tx	4	23.53	43.06
Ultrasound	11	Tx	5	17.65	38.7
Ultrasound	11	Tx	6	45.71	50.54
Ultrasound	12	Ax	1	0	0
Ultrasound	12	Ax	2	2.04	14.29
Ultrasound	12	Tx	2	0	0
Ultrasound	12	Tx	3	1.85	13.54
Ultrasound	12	Tx	4	0	0
Ultrasound	16	Ax	1	0	0
Ultrasound	16	Ax	2	0	0
Ultrasound	16	Ax	3	0	0
Ultrasound	16	Tx	1	0	0
Ultrasound	16	Tx	2	0	0
Ultrasound	16	Tx	3	8.57	28.4
Ultrasound	16	Tx	4	0	0
Ultrasound	16	Tx	5	0	0
Ultrasound	16	Tx	6	0	0
Ultrasound	19	Ax	1	93.88	24.22
Ultrasound	19	Ax	2	97.96	14.29
Ultrasound	19	Ax	3	95.97	19.73
Ultrasound	19	Tx	1	0	0
Ultrasound	19	Tx	2	94.55	22.92
Ultrasound	19	Tx	3	3.7	19.06
Ultrasound	19	Tx	4	0	0
Ultrasound	19	Tx	5	100	0

**Table 7:** Dose per participant per session, as reported by the treating SLTs and checked by the research SLT

Participant	Session no	Dose reported	Dose checked	
SS_Tx_01_F_07	4	253	378	
SS_Tx_02_M_12	1	205	266	
SS_Tx_02_M_12	2	317	321	
SS_Tx_03_M_07	3	391	368	
SS_Tx_04_M_05	5	162	163	
SS_Tx_04_M_05	6	116	119	
SS_Tx_05_M_05	4	170	176	
SS_Tx_06_F_09	2	221	224	
SS_Tx_06_M_09	1	152	160	
SS_Tx_07_M_08	1	124	139	
SS_Tx_08_M_07	2	170	212	
SS_Tx_09_M_15	1	179	253	
SS_Tx_10_F_05	2	209	211	
SS_Tx_11_M_07	4	245	159	
SS_Tx_12_F_05	2	109	116	
SS_Tx_13_F_05	3	245	258	
SS_Tx_14_F_04	4	188	213	
SS_Tx_15_M_06	5	230	279	
SS_Tx_16_F_06	5	265	277	
SS_Tx_17_F_10	4	265	330	
SS_Tx_18_M_11	6	249	235	
SS_Tx_19_M_11	2	243	242	

## Abbreviations

BCLP: Bilateral cleft lip and palate
BPVS: British Picture Vocabulary Scales
CAPS-A: Cleft Audit Protocol for Speech-Augmented
CLP: Cleft lip and palate
CONSORT: Consolidated Standards of Reporting Trials
COVID: Corona virus disease
CP: Cleft palate
CP ± L: Cleft palate ± cleft lip
CRANE: The Cleft Registry and Audit Network
DEAP: Diagnostic Evaluation of Articulation & Phonology
EPG: Elecropalatography
ESQ: Experience of Service Questionnaire
GOS.SP.ASS: Great Ormond Street Speech Assessment
ICC: Interclass correlation
ICS: Intelligibility in Context Scale
ISRCTN: International Standard Randomised Controlled Trial Number
MS: Microsoft
NHS: National Health Service
PCC: Percentage target Consonants Correct
PURE: Publication and Research
RCT: Randomised controlled trial
SD: Standard deviation
SLT: Speech and Language Therapist
UCLP: Unilateral cleft lip and palate
UK: United Kingdom
U-VBF: Ultrasound visual biofeedback

## References

[1] Bellis TH, Wohlgemuth B. The incidence of cleft lip and palate deformities in the south-east of Scotland (1971–1990). Br J Orthod. 1999;26(2):121–5. 10420246 10.1093/ortho/26.2.121

[2] Gregg TA, Leonard AG, Hayden C, Howard KE, Coyle CF. Birth prevalence of cleft lip and palate in Northern Ireland (1981 to 2000). The Cleft Palate Craniofacial Journal. 2008;45(2):141–7. 18333643 10.1597/06-045.1

[3] Medina J, Butterworth S, Fitzsimmons K, Russell C, Whaedally H, van der Meulen J: **CRANE Database 2022 Annual Report**. In*.*: Royal College of Surgeons, Clinical Effectiveness Unit; 2022.

[4] Trost JE. Articulatory additions to the classical description of the speech of persons with cleft palate. Cleft Palate J. 1981;18(3):193–203. 6941865

[5] Lee A, Gibbon FE, Spivey K. Children’s attitudes toward peers with unintelligible speech associated with cleft lip and/or palate. The Cleft Palate Craniofacial Journal. 2017;54(3):262–8. 27031270 10.1597/15-088

[6] Bessell A, Sell D, Whiting P, Roulstone S, Albery L, Persson M, Verhoeven A, Burke M, Ness AR. Speech and language therapy interventions for children with cleft palate: a systematic review. The Cleft Palate Craniofacial Journal. 2013;50(1):1–17. 10.1597/11-202 22433039

[7] Alighieri C, Bettens K, Bruneel L. D hE, Van GE, Van LK: **Effectiveness of speech intervention in patients with a cleft palate: comparison of motor-phonetic versus linguistic-phonological speech approaches**. J Speech Lang Hear Res. 2020;63(12):3909–33. 33253622 10.1044/2020_JSLHR-20-00129

[8] Hanley L, Ballard KJ, Dickson A, Purcell A. Speech intervention for children with cleft palate using principles of motor learning. Am J Speech Lang Pathol. 2023;32(1):169–89. 36475751 10.1044/2022_AJSLP-22-00007

[9] Chapman KL. Phonologic processes in children with cleft palate. The Cleft Palate Craniofacial Journal. 1993;30(1):64–72. 8418874 10.1597/1545-1569_1993_030_0064_ppicwc_2.3.co_2

[10] Cavalheiro MG, Lamônica DAC, de Vasconsellos HSR, Maximino LP. Child development skills and language in toddlers with cleft lip and palate. Int J Pediatr Otorhinolaryngol. 2019;116:18–21. 30554694 10.1016/j.ijporl.2018.10.011

[11] Chapman KL, Hardin-Jones M, Halter KA. The relationship between early speech and later speech and language performance for children with cleft lip and palate. Clin Linguist Phon. 2003;17(3):173–97. 12858838 10.1080/0269920021000047864

[12] Lee ASY, Law J, Gibbon FE: **Electropalatography for articulation disorders associated with cleft palate**. *Cochrane Database of Systematic Reviews* 2009. 10.1002/14651858.CD006854.pub2 PMC7390345 19588407

[13] Lee A: **Electropalatography**. In: *Manual of clinical phonetics.* edn.: Routledge; 2021: 339–355.

[14] Lee A, Gibbon FE, Crampin L, Yuen I, McLennan G. The national CLEFTNET project for individuals with speech disorders associated with cleft palate. Advances in Speech Language Pathology. 2007;9(1):57–64.

[15] Dokovova M, Sugden E, Cartney G, Schaeffler S, Cleland J. Tongue shape complexity in children with and without speech sound disorders. J Speech Lang Hear Res. 2023;66(7):2164–83. 37267440 10.1044/2023_JSLHR-22-00472 PMC12379088

[16] Kabakoff H, Harel D, Tiede M, Whalen DH, McAllister T. Extending ultrasound tongue shape complexity measures to speech development and disorders. J Speech Lang Hear Res. 2021;64(7):2557–74. 34232685 10.1044/2021_JSLHR-20-00537 PMC8632483

[17] Sugden E, Lloyd S, Lam J, Cleland J. Systematic review of ultrasound visual biofeedback in intervention for speech sound disorders. Int J Lang Commun Disord. 2019;54(5):705–28. 31179581 10.1111/1460-6984.12478

[18] Roxburgh Z, Cleland J, Scobbie JM, Wood SE: **Quantifying changes in ultrasound tongue-shape pre- and post-intervention in speakers with submucous cleft palate: an illustrative case study**. *Clinical Linguistics & Phonetics* 2021:1–19. 10.1080/02699206.2021.1973566 34496688

[19] Hosseinabad HH, Xing Y: **Feasibility of using ultrasound visual biofeedback to treat persistent speech sound disorders in children with cleft palate- a case series**. *Clinical linguistics & phonetics* 2024:1–32. 10.1080/02699206.2024.2306468 38282211

[20] Lewis M, Bromley K, Sutton CJ, McCray G, Myers HL, Lancaster GA: **Determining sample size for progression criteria for pragmatic pilot RCTs: the hypothesis test strikes back!** *Pilot and Feasibility Studies* 2021, **7**(1). 10.1186/s40814-021-00770-x PMC7856754 33536076

[21] Cleland J, McCluskey R, Dokovova M, Crampin L, Campbell L. A mixed-methods pilot randomised control trial of ultrasound visual biofeedback versus standard intervention for children with cleft palate +/- cleft lip: parents’ and children’s perspectives. International Journal of Speech, Language and Communication Disorders. 2024;60(1):1–16. 10.1111/1460-6984.13144 PMC11626862 39651790

[22] Cleland J, Crampin L, Campbell L, Dokovova M. Protocol for SonoSpeech Cleft Pilot: a mixed-methods pilot randomized control trial of ultrasound visual biofeedback versus standard intervention for children with cleft lip and palate. Pilot Feasibility Stud. 2022;8(1):93. 35477444 10.1186/s40814-022-01051-x PMC9043876

[23] Dunn LM. The British picture vocabulary scale. London: G L Assessment Ltd.; 2009.

[24] Dodd B, Hua Z, Crosbie S, Holm A, Ozanne A: **DEAP: diagnostic evaluation of articulation and phonology**, U.S. edition edn. San Antonio, TX: PsychCorp of Harcourt Assessment; 2006.

[25] Cleland J, Lloyd S, Campbell L, Crampin L, Palo J-P, Sugden E, Wrench A, Zharkova N. The Impact of Real-Time Articulatory Information on Phonetic Transcription: Ultrasound-Aided Transcription in Cleft Lip and Palate Speech. Folia Phoniatr Logop. 2020;72(Suppl. 2):120–30. 31129664 10.1159/000499753

[26] Sell D, Harding A, Grunwell P: **GOS.SP.ASS.'98: an assessment for speech disorders associated with cleft palate and/or velopharyngeal dysfunction (revised)**. *Int J Lang Commun Disord* 1999, **34**(1):17–33. 10.1080/136828299247595 10505144

[27] Murray E, McCabe P, Ballard KJ. A systematic review of treatment outcomes for children with childhood apraxia of speech. Am J Speech Lang Pathol. 2014;23(3):486–504. 24686844 10.1044/2014_AJSLP-13-0035

[28] Cleland J, Preston JL: **Biofeedback interventions**. In: *Interventions for Speech Sound Disorders in Children.* Second edition edn. Edited by Williams LA, McLeod S, McCauley RJ. Baltimore, MD: Paul H. Brookes Publishing Co.; 2021: 573–600.

[29] Cleland J, Wrench A, Lloyd S, Sugden E. ULTRAX2020: ultrasound technology for optimising the treatment of speech disorders : clinicians’ resource manual. In. Glasgow: University of Strathclyde; 2018.

[30] Articulate Instruments L: **SonoSpeech: ultrasound application for recording, client assessment and visual feedback**. In*.* Edinburgh, UK: Articulate Instruments; 2019.

[31] Bowen DJ, Kreuter M, Spring B, Cofta-Woerpel L, Linnan L, Weiner D, Bakken S, Kaplan CP, Squiers L, Fabrizio C, et al. How we design feasibility studies. Am J Prev Med. 2009;36(5):452–7. 19362699 10.1016/j.amepre.2009.02.002 PMC2859314

[32] Patrick K, Fricke S, Rutter B, Cleland J: **Clinical application of usage-based phonology: treatment of cleft palate speech using usage-based electropalotography**. *International Journal of Speech-Language Pathology* 2023:1–16. 10.1080/17549507.2023.2238924 37652151

[33] McLeod SH, Linda J. McCormack, Jane **The intelligibility in Context Scale: validity and reliability of a subjective rating measure**. J Speech Lang Hear Res. 2012;55(2):648–56. 22215036 10.1044/1092-4388(2011/10-0130)

[34] Mirihagalla Kankanamalage I, Cleland J, Cohen W: **Translation and validation of the Intelligibility in Context Scale into Sinhala for adolescents in Sri Lanka with cleft lip and palate**. *Clinical Linguistics & Phonetics* 2022, **0**(0):1–17. 10.1080/02699206.2022.2120417 36093956

[35] Seifert M, Davies A, Harding S, McLeod S, Wren Y: **Intelligibility in 3-year-olds with cleft lip and/or palate using the intelligibility in Context Scale: findings from the cleft collective cohort study**. *The Cleft Palate-Craniofacial Journal* 2021:105566562098574. 10.1177/1055665620985747 33530712

[36] Klassen AF, Riff KWW, Longmire NM, Albert A, Allen GC, Aydin MA, Baker SB, Cano SJ, Chan AJ, Courtemanche DJ, et al. Psychometric findings and normative values for the CLEFT-Q based on 2434 children and young adult patients with cleft lip and/or palate from 12 countries. Can Med Assoc J. 2018;190(15):E455–62. 29661814 10.1503/cmaj.170289 PMC5903887

[37] Brown A, Ford T, Deighton J, Wolpert M. Satisfaction in child and adolescent mental health services: translating users’ feedback into measurement. Adm Policy Ment Health. 2014;41(4):434–46. 22829193 10.1007/s10488-012-0433-9

[38] Sell D, John A, Harding-Bell A, Sweeney T, Hegarty F, Freeman J. Cleft audit protocol for speech (CAPS-A): a comprehensive training package for speech analysis. Int J Lang Commun Disord. 2009;44(4):529–48. 18821108 10.1080/13682820802196815

[39] Wickham H, Averick M, Bryan J, Chang W, McGowan LDA, François R, Grolemund G, Hayes A, Henry L, Hester J, et al. Welcome to the Tidyverse. Journal of Open Source Software. 2019;4(43):1686.

[40] R core team: **R: a language and environment for statistical computing**. In*.* Vienna, Austria: R Foundation for Statistical Computing; 2023.

[41] Wickham H: **ggplot2: elegant graphics for data analysis**; 2016.

[42] Kassambara A: **ggpubr:** ‘**ggplot2**’ **based publication ready plots**. In*.*, 0.6.0 edn; 2023.

[43] Revelle W: **psych: procedures for psychological, psychometric, and personality research**. In*.*, 2.4.1 edn. Evanston, Illinois: Northwestern University; 2024.

[44] Koo TK, Li MY. A guideline of selecting and reporting intraclass correlation coefficients for reliability research. J Chiropr Med. 2016;15(2):155–63. 27330520 10.1016/j.jcm.2016.02.012 PMC4913118

[45] Pennington L, Rauch R, Smith J, Brittain K. Views of children with cerebral palsy and their parents on the effectiveness and acceptability of intensive speech therapy. Disabil Rehabil. 2020;42(20):2935–43. 30925074 10.1080/09638288.2019.1577504

[46] Sim J. Should treatment effects be estimated in pilot and feasibility studies? Pilot and Feasibility Studies. 2019;5:107. 31485336 10.1186/s40814-019-0493-7 PMC6712606

[47] Patrick K, Cleland J, Rutter B, Fricke S: **Gradient speech change during intervention for school-aged children and adults with cleft palate +/- lip**. *Clinical Linguistics & Phonetics* 2024:1–32. 10.1080/02699206.2024.2355472 38869102

[48] Cleland J, Scobbie JM. The dorsal differentiation of velar from alveolar stops in typically developing children and children with persistent velar fronting. J Speech Lang Hear Res. 2021;64(6S):2347–62. 33719530 10.1044/2020_JSLHR-20-00373

[49] Cleland J, McCluskey R, Dokovova M, Crampin L, Campbell L: **Clinicians’ perspectives on using ultrasound visual biofeedback for research and practice with people with cleft palate ± cleft lip**. *The Cleft Palate Craniofacial Journal* (in press). 10.1177/10556656251322279 40223291

